# Effects of dietary vitamin D supplementation on bone microarchitecture, mineralization, and mechanical properties in Wistar rat animal model

**DOI:** 10.1038/s41598-026-41077-2

**Published:** 2026-02-22

**Authors:** Cezary Osiak-Wicha, Siemowit Muszyński, Katarzyna Kras, Ewa Tomaszewska, Beata Szymczyk, Maria Oczkowicz, Małgorzata Świątkiewicz, Dariusz Wiącek, Daniel Kamiński, Marcin B. Arciszewski

**Affiliations:** 1 Department of Animal Anatomy and Histology, Faculty of Veterinary Medicine, University of Life Sciences in Lublin, Akademicka 12, 20-950 Lublin, Poland; 2 Department of Biophysics, Faculty of Environmental Biology, University of Life Sciences in Lublin, Akademicka 13, 20-950 Lublin, Poland; 3 Department of Animal Physiology, Faculty of Veterinary Medicine, University of Life Sciences in Lublin, Akademicka 12, 20-950 Lublin, Poland; 4 Department of Animal Nutrition and Feed Science, National Research Institute of Animal Production, Krakowska 1, 32-083 Balice, Poland; 5 Department of Animal Molecular Biology, National Research Institute of Animal Production, Krakowska 1, 32-083 Balice, Poland; 6 Bohdan Dobrzański Institute of Agrophysics of the Polish Academy of Sciences, Doświadczalna 4, 20-290 Lublin, Poland; 7 Department of Organic and Crystallochemistry, Institute of Chemical Sciences, Faculty of Chemistry, Maria Curie-Sklodowska University, M. Curie-Sklodowskiej 3, 20-031 Lublin, Poland

**Keywords:** Mineral homeostasis, Bone remodeling, Bone metabolism, Growth plate, Bone markers, Biochemistry, Endocrinology, Health care, Medical research, Physiology

## Abstract

Vitamin D is essential for proper skeletal development, yet the specific dose-dependent effects of its dietary supplementation during the transition from late adolescence through young adulthood remain unclear. Because the standard diet already supplies 1,000 IU/kg vitamin D₃ and is widely regarded as adequate for growing rats, poorer skeletal outcomes at 0 IU/kg and favorable outcomes at 1,000 IU/kg are expected. The studies objective was therefore to quantify the magnitude and map which dimensions of bone quality respond to vitamin D across the adolescent-to-young-adult transition, and to test whether a supraphysiological intake (5,000 IU/kg) confers additional benefit and we hypothesized that moderate vitamin D would improve bone outcomes versus deficiency, whereas a high dose would offer no added advantage. Male and female Wistar rats (≈ 7 weeks old; *n* = 6/sex/group) were fed for 12 weeks diets containing 0, 1000, or 5000 IU/kg vitamin D. The right femur was assessed by DXA (BMD), three-point bending (strength, energy absorption, stiffness), growth-plate morphometry, elemental composition, and X-ray diffraction of hydroxyapatite; RANKL/OC/VEGF immunoexpression and serum 25(OH)D were measured. Diets produced graded systemic exposure: 25(OH)D increased with dose in both sexes. Across outcomes, sex showed a strong main effect (males larger/stronger), while dose effects were selective. Compared with 0 IU/kg, 1000 IU/kg increased BMD and several mechanical endpoints (e.g., maximum force/energy) primarily in males, and improved material behavior (post-yield strain) without altering elastic modulus. Growth plate effects were limited: the hypertrophic (Zone IV) thickness was lower with deficiency and greater with supplementation, with sex- and dose-specific patterns; other zones showed minimal dose effects. The 5000 IU/kg diet raised 25(OH)D but did not consistently outperform 1000 IU/kg and showed no broad detriments across measured variables. During late growth, moderate vitamin D (1000 IU/kg) improves femoral mass and selected mechanical/biomechanical properties relative to deficiency, with sex-specific magnitudes of effect. A higher dose (5000 IU/kg) increases circulating vitamin D metabolite, but provides no clear additional skeletal benefit. These data support moderate supplementation as optimal in this model.

## Introduction

Vitamin D, a vital fat-soluble compound, plays an essential role in maintaining skeletal health across animal species. It is primarily synthesized in the skin through ultraviolet B (UVB) radiation, with additional contributions from dietary sources such as fatty fish, fortified foods, and supplements^1^. Once absorbed or synthesized, vitamin D undergoes two hydroxylation steps; first in the liver to form 25-hydroxyvitamin D [25(OH)D], and then in the kidneys to produce the active form, 1,25-dihydroxyvitamin D [1,25(OH)₂D], or calcitriol^2^. Through binding to vitamin D receptors (VDRs), calcitriol regulates critical physiological processes, including calcium and phosphorus metabolism, thus promoting bone health. Vitamin D’s role extends beyond mineral absorption, influencing the production of proteins such as osteocalcin (OC) and receptor activator of nuclear factor kappa-Β ligand (RANKL), which are vital for bone turnover and structural integrity^3^.

Vitamin D is indispensable for proper bone formation and maintenance by enhancing intestinal absorption of calcium and phosphorus, essential minerals for bone matrix mineralization. A complete deprivation of vitamin D during growth is well-known to impair mineralization, leading to classic signs of rickets, such as widened growth plates, reduced trabecular bone volume, decreased ash content, and accumulation of unmineralized matrix^4–7^. However, it is increasingly recognized that when dietary calcium and phosphorus intake are adequate, a restriction of vitamin D, even below standard requirements, may not immediately lead to significant impairments in bone mass, mineral density, or mechanical performance^8,9^. Thus, the skeletal consequences of vitamin D deficiency are modulated by the broader nutritional environment, particularly mineral availability.

In contrast, the effects of supraphysiological doses of vitamin D, commonly defined as intakes considerably above physiological requirements, remain more ambiguous. Some studies, such as those performed in IL-10 knockout (IL-10⁻/⁻) mice receiving 5000 IU/kg, reported no significant skeletal differences compared to standard supplementation^9^. Similarly, very high doses (e.g., 40,000 IU/kg) have shown modest positive effects on bone mechanics, but on older rats and only in specific experimental conditions involving additional physiological stress^10,11^. Moderate high doses, such as 10,000 IU/kg, have been associated with beneficial effects on bone trabecular structure in growing mice^12^. Overall, available evidence suggests that when calcium intake is sufficient and no additional physiological stress is present, vitamin D supplementation above adequacy thresholds often does not further improve bone quality in healthy growing rodents^13^. Importantly, even at high levels, vitamin D has generally not induced pathological skeletal changes in young rodents, supporting the concept of a relatively broad physiological plateau for vitamin D effects on bone^9,12^.

Despite extensive research on vitamin D and bone health, there remains a significant gap in studies focusing specifically on the late adolescent period in rats (approximately 7 to 19 weeks of age). Most available studies have either targeted early post-weaning stages (3–8 weeks) to model rickets or have assessed adult skeletal health (> 5 months) to study osteomalacia and bone loss^6,14^. The period corresponding to late growth and the final phase of peak bone mass (PBM) development is less well-characterized, even though it is crucial for long-term skeletal growth. Previous research has often been limited to isolated assessments of bone mineral density (BMD) or serum biochemical markers, without integrating broader tissue-level assessments that reflect the structural, mechanical, and molecular complexity of skeletal adaptation.

The present study aims to address these gaps by investigating the dose-dependent effects of dietary vitamin D supplementation (0, 1000 IU/kg, and 5000 IU/kg) on skeletal development in Wistar rats during the critical late adolescent growth phase. In addition to conventional assessments of BMD, this research integrates detailed evaluations of trabecular and cortical bone microstructure, mechanical strength, material properties, macro- and microelement composition, hydroxyapatite crystallographic features, and immunohistochemical reactivity of key regulators of bone metabolism.

Given the established role of vitamin D in calcium–phosphate metabolism, endochondral ossification, and bone remodeling, we hypothesized that graded dietary vitamin D₃ (0, 1000, 5000 IU/kg) would differentially affect femoral growth and mineralization in adolescent-to–young adult Wistar rats, and that these effects would depend on sex. Specifically, we expected that vitamin D₃ deficiency (0 IU/kg) would impair somatic and femoral outcomes compared with supplemented diets, that the established AIN-93G level (1000 IU/kg) would support optimal femoral geometry, mineralization, and mechanical performance, and that a higher intake (5000 IU/kg) would not produce consistent additional improvements. We further hypothesized that dose- and sex-related changes in femoral structure would be paralleled by shifts in serum markers and by immunoexpression of key regulators of bone metabolism, reflecting systemic and local responses to vitamin D status. These hypotheses were tested in a two-factor design with vitamin D dose and sex as fixed factors. Through a multidimensional and integrative methodology, this work provides a more detailed characterization of vitamin D–bone relationships than is typical in the field. Our emphasis is not on reestablishing known dose effects but on mapping how vitamin D status shapes distinct dimensions of femoral quality across the adolescent-to–young-adult transition, thereby offering measured, evidence-based context for nutritional strategies in laboratory and livestock animals.

## Materials and methods

### Animals

The experiment was conducted at the Experimental Station of the National Research Institute of Animal Production in Balice. All experimental procedures were reviewed and approved by the 2nd Local Ethics Committee for Animal Experimentation in Krakow (approval no. 136/2018) and complied with the ARRIVE guidelines. A total of thirty-six Wistar rats (18 males and 18 females), obtained from the Centre for Experimental Medicine of the Medical University of Silesia in Katowice (Poland), were used in the study. At the beginning of the experiment, the rats were approximately seven weeks old. Animals were housed individually in steel cages under controlled environmental conditions (room temperature 23 ± 2 °C, 12-hour light/dark cycle, relative humidity 40%–55%). Throughout the 10-day adaptation period and the subsequent 12-week experimental phase, the rats had *ad libitum* access to feed and water. Rats were allocated by stratified randomization to three diet groups (*n* = 12 per group; 6 males, 6 females). Stratification factors were sex and baseline body mass. Within each sex, animals were ranked by body mass and divided into tertiles; within each tertile, rats were assigned using simple random allocation to 0, 1000, or 5000 IU/kg diets in a 1:1:1 ratio. Body weight and feed intake were recorded weekly. The first group (Group I) received a modified AIN-93G experimental diet^15^, which in its original formulation includes vitamin D₃ at 1 IU/g (1,000 IU/kg). The modification was by omitting vitamin D₃ from the vitamin premix (target 0 IU/kg). Two supplemented diets were then formulated at 1,000 IU/kg (Group II; equivalent to the original AIN-93G level) and 5,000 IU/kg (Group III) by adding cholecalciferol to the base mix. At the end of the 12-week exposure period, all animals were euthanized using CO₂ in an Easy-Box euthanasia system (Minerve, U.K.). Euthanasia was performed using 100% CO₂ delivered at a rate of approximately 20% of the cage volume per minute (~ 3.2 L/min for a 16-liter cage), as recommended by the AVMA Guidelines for the Euthanasia of Animals (2020)^16^. Gas was administered continuously for at least one minute following cessation of respiration to ensure death. Following euthanasia, blood was collected from the heart using a sterile syringe, and both the right and left femur from each animal were harvested, wrapped in sterile gauze moistened with 0.9% saline, sealed in airtight bags to prevent desiccation, and stored at − 20 °C for further analysis. All procedures were performed in accordance with the national and international guidelines and regulations, including the Polish Act on the Protection of Animals Used for Scientific or Educational Purposes (Dz.U. 2015 poz. 266)^17^ and the EU Directive 2010/63/EU^18^.

### Bone analysis

The right femur from each animal was used for bone analysis. After thawing overnight at 4 °C, bone mineral density (BMD) was measured using a Lunar densitometer (GE, Madison, WI, USA) with the dual-energy X-ray absorptiometry (DXA) method. Each femur was scanned along its entire length, and BMD values of whole bones were recorded for analysis. Following DXA assessment, femurs underwent biomechanical testing using a three-point bending test to evaluate their structural properties. Testing was performed on a Zwick Z010 universal testing machine (Zwick-Roell GmbH & Co., Ulm, Germany), with the span distance of 14 mm (ca. 40% of mean bone length) and a constant loading rate of 10 mm/min. Bones were positioned horizontally on two supports, and load was applied at the mid-diaphysis. During testing, load–displacement curves were generated and used to determine the following mechanical parameters: yield load (F_yield_), fracture load (F_max_), stiffness, elastic work (W_yield_), and work to fracture (W_max_). Mechanical data were analyzed using Origin software (version 2022, OriginLab, Northampton, MA, USA). After mechanical testing, femurs were transversely sectioned at the mid-diaphysis using a diamond bandsaw (MBS 240/E, Proxxon GmbH, Foehren, Germany). Based on the load–deformation curves and geometric properties, apparent material properties were calculated from whole-bone bending using standard beam-theory equations, including yield strain (Ɛ_yield_), breaking strain (Ɛ_max_), Young’s modulus, yield stress (σ_yield_), and breaking stress (σ_max_). All procedures followed validated protocols for biomechanical and material property analysis in bone research^19^.

Following testing, bone mid-diaphysis were collected, bones were thoroughly cleaned, defatted (2:1, v: v chloroform: methanol), and calcined in a muffle furnace at 500 °C for 24 h to obtain bone ash. The resulting ash was finely ground and prepared for X-ray diffraction analysis and macro- and microelements measurements.

### Immunohistochemistry, growth plate, and metaphyseal trabecular morphology

Fragments of the left femurs containing the growth plate were separated from the remaining bone using a diamond band saw MBS 240/E (Proxxon GmbH, Foehren, Germany). Samples were fixed in 4% buffered formalin for 24 h, then decalcified in 10% buffered EDTA (pH 7.4) for 3 weeks on a shaker at 4 °C, dehydrated in an ascending ethanol series, fixed with xylene, and embedded in paraffin. Sagittal Sect.  3 μm thick were cut using a rotary microtome (Microm HM 360, Microm, Walldorf, Germany).

Immunohistochemistry (IHC) was performed using antibodies against RANKL (E-AB-30151, Elabscience, Wuhan, China, dilution 1:100), OC (ab13420, Abcam, Cambridge, UK, dilution 1:100) and VEGF (orb191500, Biorbyt, Cambridge, UK, dilution 1:100) according to a previously described protocol^20^. On the first day, enzymatic epitope retrieval was carried out with proteinase K (Sigma-Aldrich, St. Louis, MO, USA; 20 µg/ml in Tris-HCl buffer, 37 °C) for 10 min, followed by a 10-minute incubation in 3% H₂O₂ for blocking endogenous peroxidase activity. After blocking nonspecific binding sites for 5 min (UltraVision Protein Block, Thermo Scientific, Waltham, MA, USA), the samples were incubated overnight at 4 °C with primary antibodies. On the second day, the BrightVision detection system (poly-HRP-anti-Ms/Rb IgG, ImmunoLogic WellMed B.V., Duiven, Netherlands) was applied for 45 min in total, and immunostaining was performed using 3,3′-diaminobenzidine (DAB substrate kit; ab64238; Abcam, Cambridge, UK). Between each step, slides were rinsed with PBS. After DAB staining, the sections were washed with distilled water, counterstained with Mayer’s hematoxylin (Patho, Mar-Four, Konstantynów Łódzki, Poland), washed with tap water for 10 min, dehydrated in ascending concentrations of ethanol, cleared in xylene, mounted with Shandon Consul-Mount (Thermo Scientific, Waltham, MA, USA), and dried at 37 °C for 12 h. The specificity of the antibodies was evaluated using a negative control (no primary antibody) and a preabsorption test with an excess of the target protein. These controls were performed simultaneously using the same protocol. No positive immunoreactivity was observed in the control stainings.

The stained slides were examined under a light microscope (CX43, Olympus, Tokyo, Japan). High-resolution digital photographs were taken using Cell^M 2.3 software (Olympus) under consistent lighting conditions and uniform settings for brightness and contrast. All sections were processed in a single batch with identical reagent lots where possible, identical incubation times, and a fixed DAB development time; imaging was performed in one session per antibody at fixed magnification and identical camera/exposure/white-balance settings, verified with a calibration slide.

For histomorphometric evaluation of the growth plate, sections were stained using hematoxylin and eosin (H&E). Digital images were captured with an Olympus BX53 microscope equipped with a DP74 camera (Olympus, Tokyo, Japan) and analyzed using Olympus cellSens software, version 1.5 (Olympus, Tokyo, Japan). The thickness of individual histological zones within the growth plate was measured according to established morphological criteria. The reserve zone (Zone I) consisted of single or paired chondrocytes surrounded by abundant extracellular matrix. The proliferative zone (Zone II) contained flattened chondrocytes arranged in longitudinal columns undergoing mitotic division. The hypertrophic zone (Zone III) was characterized by a marked increase in chondrocyte size and a less regular columnar arrangement, while the ossification zone (Zone IV) represented the transition region between cartilage and bone, showing chondrocyte degeneration and matrix mineralization^21^. Measurements were performed at four equidistant sites along the growth plate, and the mean thickness for each zone was calculated for every animal. All analyses were conducted by a single blinded observer to ensure consistency and minimize bias^22^.

Metaphyseal trabecular architecture was quantified on adjacent sections stained with Safranin O or Picrosirius Red. Images were acquired under polarized light (Picrosirius Red) using the microscopy system described above, and analyzed in ImageJ^23^ (er. 1.54f, National Institutes of Health, Bethesda, MD, USA; available at: https://imagej.net/ij/index.html). A standardized metaphyseal region of interest (ROI) was placed 0.3 mm distal to the growth plate, immediately within secondary spongiosa (excluding the primary spongiosa adjacent to the cartilage), avoiding cortical bone and primary spongiosa. The following 2D histomorphometric parameters were reported: bone volume fraction (bv/tv), trabecular number, trabecular thickness, and trabecular separation. To ensure reproducibility, three non-overlapping fields were evaluated on three sections per animal at fixed magnification, with random field selection and coded filenames to maintain blinding.

### Immunoexpression of bone markers

The imunoexpression levels of RANKL, OC, and VEGF were evaluated by quantification of staining intensity using ImageJ software version 1.54f (NIH, Bethesda, MD, USA)^23^, following the method described by Cizkova et al. (2021)^24^. First, the images were processed using the IHC Profiler plugin^25^, with color deconvolution applied to isolate the DAB staining and convert it into 8-bit greyscale. ROIs were selected in three predefined compartments: trabecular bone osteocytes (individual osteocyte profiles within secondary trabeculae, avoiding empty lacunae and marrow spaces), growth-plate matrix (interterritorial matrix within the hypertrophic zone, avoiding cell nuclei and calcified cartilage bars) and compact bone (interstitial/lamellar matrix in mid-cortex, avoiding Haversian/Volkmann canals, cement lines, and periosteal/endosteal edges). ROI placement used a stratified-random approach within each compartment at fixed microscope settings; ROI size was kept constant using ImageJ ROI templates. For each marker and anatomical region, 30 measurements were taken per specimen, and the mean value was used for statistical analysis. Staining intensity was recorded as mean gray value (scale: 0 = black, 255 = white), and converted into optical density (OD) using the formula:

$${\mathrm{OD}}\,=\, - \,{\mathrm{log}}\left( {{\mathrm{255}}/{\mathrm{x}}} \right)$$

where *x* is the mean gray value. OD thresholds were used to categorize the staining: < 0.2 = negative, 0.2–0.4 = weak, 0.4–0.6 = moderate, and > 0.6 = strong^24^. All measurements were performed by the same investigator, who was blinded to the treatment allocation of the samples.

### Serum biomarkers analysis

Blood serum concentrations of bone metabolism and inflammatory biomarkers were determined using commercial rat-specific enzyme-linked immunosorbent assay (ELISA) by Elabscience (Houston, TX, USA) according to the manufacturer’s protocols. The assays were growth hormone (GH, E-EL-R0029), osteocalcin (OC, E-EL-R0243), osteoprotegerin (OPG, E-EL-R0050), bone alkaline phosphatase (BALP, E-EL-R0113), receptor activator of nuclear factor kappa B ligand (RANKL, E-EL-R0841), insulin-like growth factor-1 (IGF-1, E-EL-R0010), interleukin 1 (IL-1, E-EL-R0012), interleukin 6 (IL-6, E-EL-R0015), interleukin 10 (IL-10, E-EL-R0016), and tumor necrosis factor alpha (TNF- α, E-EL-R2856). Measurements were performed in two technical replicates. The intra-assay coefficient of variation for all kits was < 7%. Each sample was analyzed in triplicate on a Benchmark Plus microplate spectrophotometer (Bio-Rad Laboratories, Hercules, CA, USA).

### X-ray diffraction analysis

To assess the crystallographic properties of bone mineral, X-ray diffraction (XRD) analysis was performed on the bone ash samples. XRD measurements were conducted using a high-resolution Empyrean X-ray diffractometer (PANalytical, Almelo, The Netherlands) equipped with a Cu Kα radiation source (λ = 1.5478 Å) and Ni filter, operating at 40 kV and 30 mA, with radiation detected by a proportional detector. Samples were scanned in θ–2θ geometry over a 2θ range of 10° to 80°, with a step size of 0.01° and a counting time of 6 s per point. Measurements were performed at room temperature, using a source divergence slit and detector slit of 1/8°, with Soller slits applied. Bragg peaks and crystallographic planes were identified using the HighScore Plus software package (PANalytical, Almelo, The Netherlands). The peak position and full width at half maximum (FWHM) were calculated by fitting the diffraction (002) peaks to the Voigt function using OriginPro 2020 software (OriginLab, Northampton, MA, USA). The crystallite size (Dp) was calculated using the Scherrer equation, incorporating a shape constant of 0.9 and an instrumental broadening correction of 0.01°. Assuming hexagonal hydroxyapatite (P6₃/m), interplanar spacings dₕₖₗ were obtained from Bragg’s law. The lattice parameters were calculated from selected reflections: *c* from the 002 peak (c = 2·d₀₀₂) and *a* from the (310) reflection using the hexagonal relation 1/d² = (4/3)(h² + hk + k²)/a² + l²/c², which for the (310) yields a = d₃₁₀·√(52/3)^26,27^.

### Macro- and microelements analysis

The elemental composition of the bone mineral phase was determined using inductively coupled plasma optical emission spectrometry (ICP-OES) on bone ash samples. Elemental quantification was performed with an iCAP Series 6500 ICP-OES spectrometer (Thermo Scientific, Waltham, MA, USA). A TraceCERT multi-element stock solution (Sigma-Aldrich, St. Louis, MO, USA) was used to prepare calibration standards. Elements analyzed included calcium (Ca), phosphorus (P), magnesium (Mg), copper (Cu), iron (Fe), and manganese (Mn). All measurements were conducted in triplicate to ensure analytical precision.

### Statistical analysis

All analyses were conducted in GraphPad Prism 10.5.0 for Windows (GraphPad Software, San Diego, CA, USA). Assumptions were evaluated on model residuals using the Shapiro-Wilk test for normality and Levene’s test for homogeneity of variances. When assumption checks indicated material departures, variance-stabilizing transformations were applied. A two-way factorial ANOVA with fixed factors vitamin D dose (0, 1000, 5000 IU/kg) and sex (male, female) was fit. When the dose×sex interaction was significant (*p* < 0.05), main effects were not interpreted; instead, simple effects from the same model were examined (dose within each sex; sex within each dose) using estimated marginal means with Tukey adjusted pairwise comparisons. When the interaction was not significant, main effects from the factorial model were interpreted and Tukey post hoc comparisons were performed across levels as appropriate. Statistical significance was set at *p* < 0.05. Data are presented as mean ± standard error of the mean (SEM), and model estimates are reported as estimated marginal means with 95% confidence intervals.

## Results

### Somatic and bone parameters

**Fig. 1:**
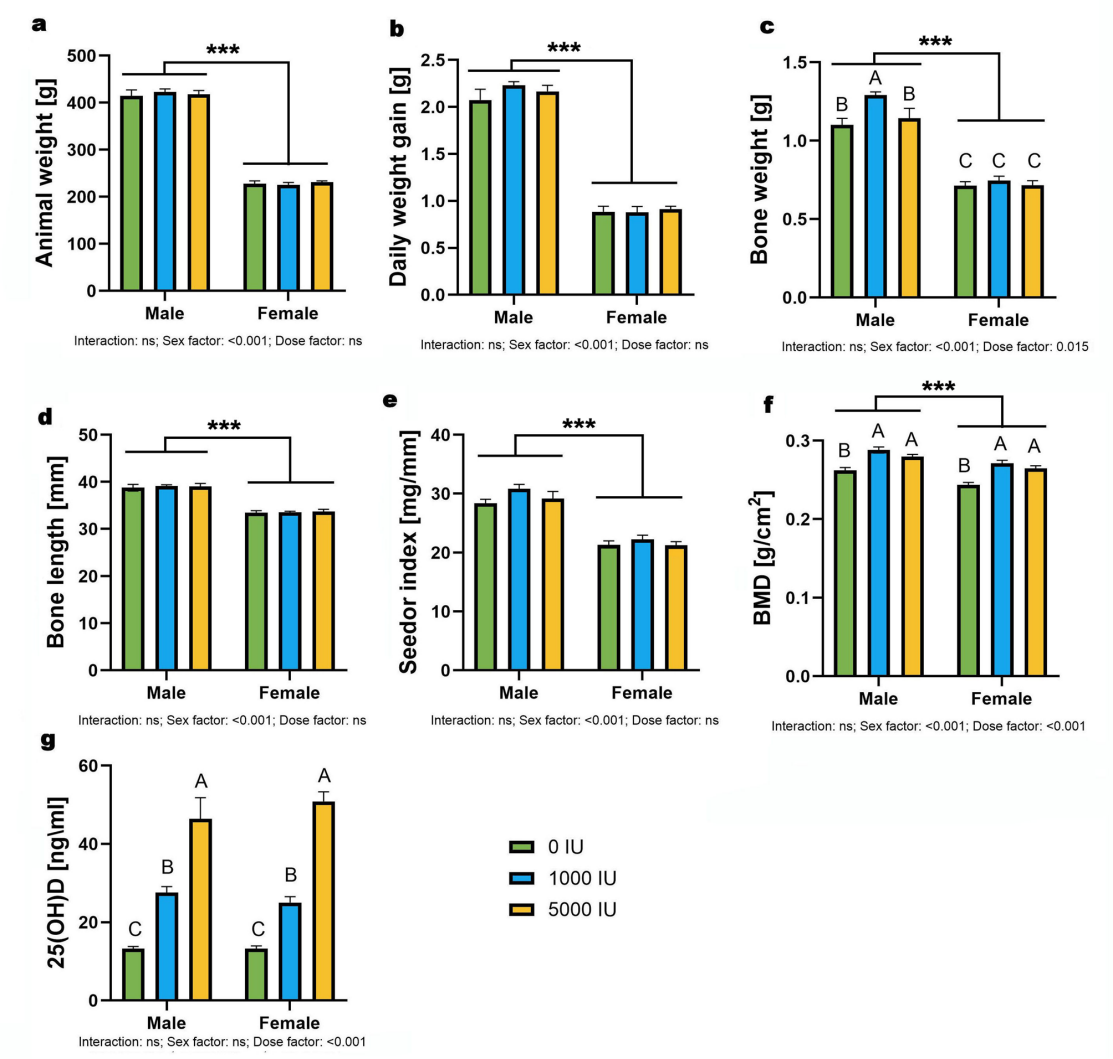
Effects of dietary vitamin D supplementation on somatic and bone parameters in male and female Wistar rats. Animals were assigned to one of three dietary groups: 0 IU/kg (Group I), 1000 IU/kg (Group II), or 5000 IU/kg (Group III) of vitamin D₃ (*n* = 6 per sex per group): (**a**) final body weight, (**b**) average daily weight gain, (**c**) bone weight, (**d**) bone length, (**e**) Seedor index, (**f**) bone mineral density and (**g**) serum 25(OH)D₃ concentration. Data are presented as mean ± SEM. Statistical analyses were performed using GraphPad Prism (version 10.5.0), with two-way ANOVA used to assess the effects of vitamin D dose, sex, and their interaction. Statistical significance was set at *p* < 0.05. Capital letters indicate significant differences between dietary doses within the sex groups; brackets denote significant differences between sexes; and asterisks (*) represent p-values (***p < 0.001).

Body weight and daily weight gain showed no dose×sex interaction and no dose effect; there was a strong sex effect (*p* < 0.001), with males heavier and gaining more than females across groups (Fig. 1a–b). Bone mass showed main effects of sex (*p* < 0.001) and dose (*p* = 0.015) without interaction; males exceeded females overall, and pooled across sex the 1000 IU/kg group was higher than 0 IU/kg and 5000 IU/kg, with no other dose differences (Fig. 1c). Bone length and Seedor index showed no interaction and only a sex effect (both *p* < 0.001); males exceeded females, with no dose effect (Fig. 1d–e). BMD exhibited main effects of sex and dose (both *p* < 0.001) without interaction; males exceeded females overall, and pooled across sex the deficient group had lower BMD than both supplemented groups, which did not differ from each other (Fig. 1f). Serum 25(OH)D showed a dose effect only (*p* < 0.001) with graded increases from 0 to 1000 to 5000 IU/kg in both sexes (Fig. 1g).

### Mechanical and geometrical properties

**Fig. 2:**
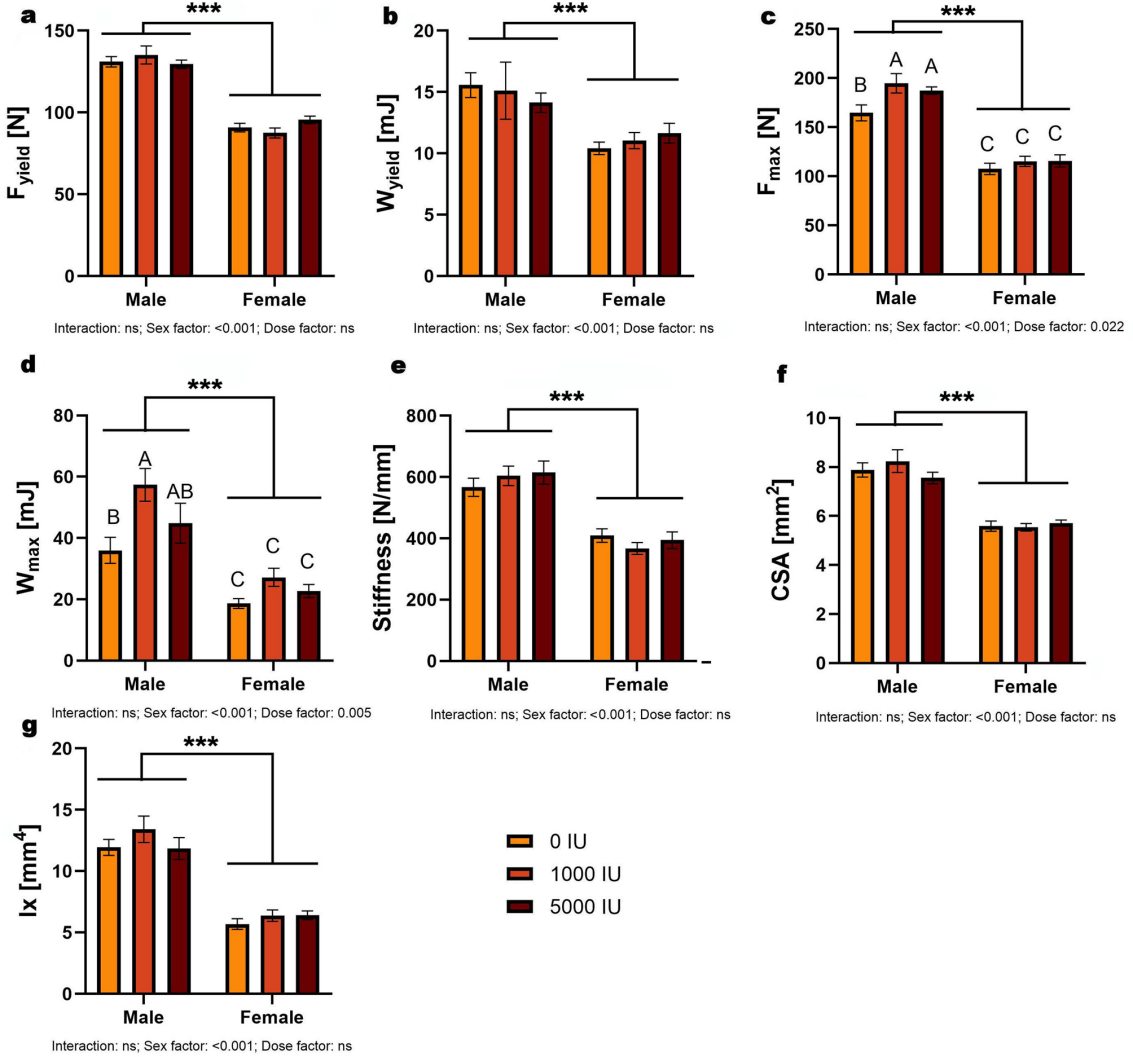
Effects of dietary vitamin D supplementation on mechanical and geometrical bone properties in male and female Wistar rats. Animals were assigned to one of three dietary groups: 0 IU/kg (Group I), 1000 IU/kg (Group II), or 5000 IU/kg (Group III) of vitamin D₃ (*n* = 6 per sex per group): (**a**) yield force, (**b**) elastic work, (**c**) breaking force, (**d**) work to fracture, (**e**) stiffness, (**f**) cross-sectional area and (**g**) moment of inertia. Data are presented as mean ± SEM. Statistical analyses were conducted in GraphPad Prism (version 10.5.0) using two-way ANOVA to assess the effects of vitamin D dose, sex, and their interaction. Statistical significance was set at *p* < 0.05. Capital letters indicate significant differences between dietary doses within the sex groups; brackets denote significant differences between sexes; and asterisks (*) represent p-values (***p < 0.001).

F_yield_ and W_yield_ showed no dose×sex interaction and no dose effect; both exhibited a strong sex effect (*p* < 0.001), with males higher than females overall (Fig. 2a–b). F_max_ showed main effects of sex (*p* < 0.001) and dose (*p* = 0.022) without interaction; males exceeded females overall, and pooled across sex the deficient group was lower than both supplemented groups, which did not differ (Fig. 2c). W_max_ likewise showed main effects of sex (*p* < 0.001) and dose (*p* = 0.005) without interaction; males exceeded females overall, and pooled across sex the 0 IU/kg group was lower than 1000 IU/kg (other pairwise comparisons not significant; Fig. 2d). Stiffness showed a sex effect only (*p* < 0.001) with males higher (Fig. 2e). Cross-sectional area and moment of inertia showed sex effects only (both *p* < 0.001), with no dose or interaction effects (Fig. 2f–g).

### Apparent bone material properties

**Fig. 3:**
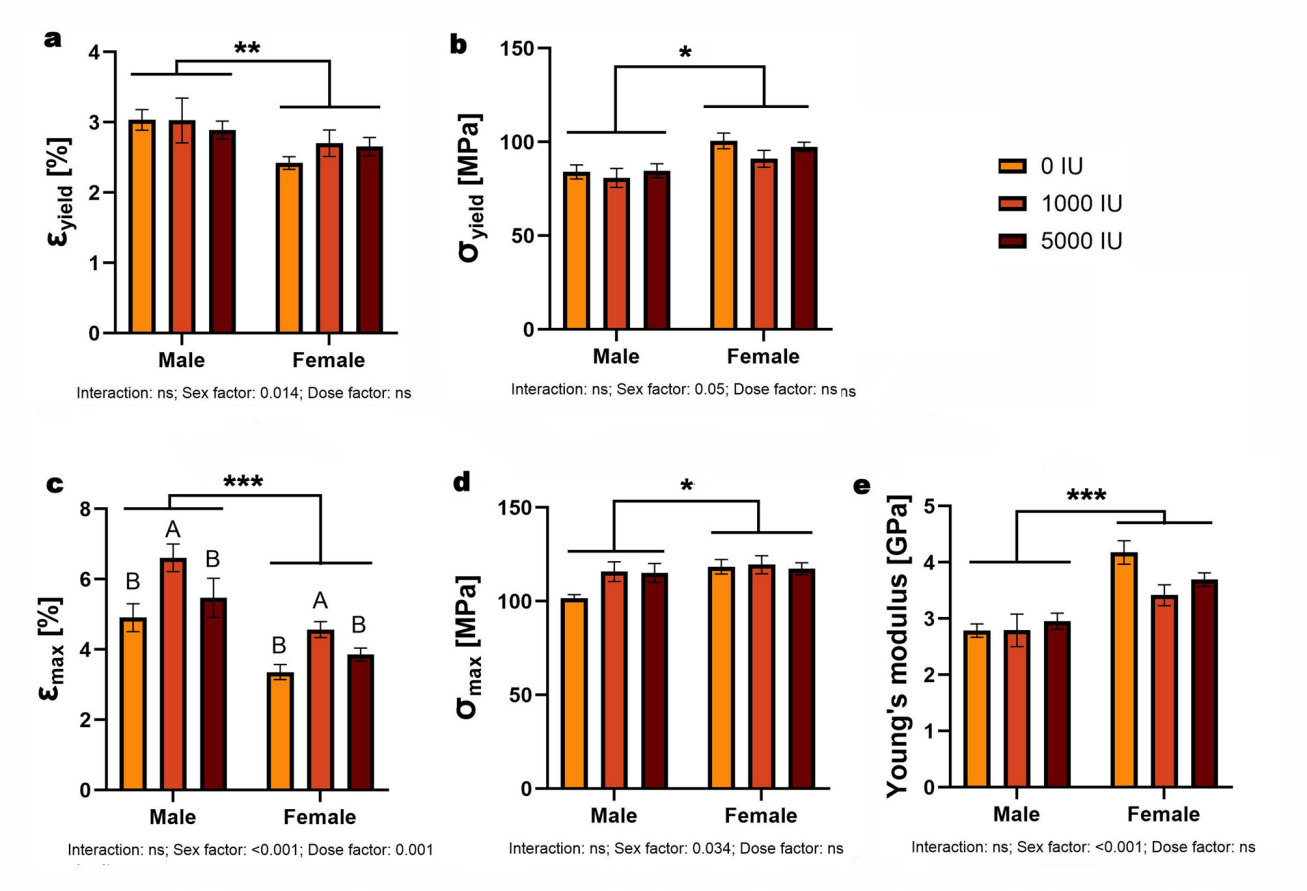
Effects of dietary vitamin D supplementation on apparent bone material properties in male and female Wistar rats. Animals were assigned to one of three dietary groups: 0 IU/kg (Group I), 1000 IU/kg (Group II), or 5000 IU/kg (Group III) of vitamin D₃ (*n* = 6 per sex per group): (**a**) yield strain, (**b**) yield stress, (**c**) breaking strain, (**d**) breaking stress, and (**e**) Young’s modulus. Data are presented as mean ± SEM. Statistical analyses were conducted in GraphPad Prism (version 10.5.0) using two-way ANOVA to assess the effects of vitamin D dose, sex, and their interaction. Statistical significance was set at *p* < 0.05. Capital letters indicate significant differences between dietary doses within the sex groups; brackets denote significant differences between sexes; and asterisks (*) represent p-values (*p < 0.05; **p < 0.01; ***p < 0.001).

ε_yield_ showed no dose×sex interaction and no dose effect; there was a sex effect (*p* = 0.014), with males higher than females overall (Fig. 3a). σ_yield_ showed a sex effect only (*p* < 0.001), with females higher than males overall (Fig. 3b). ε_max_ showed main effects of sex (*p* < 0.001) and dose (*p* = 0.001) without interaction; males exceeded females overall, and pooled across sex the 1000 IU/kg group was higher than 0 and 5000 IU/kg (Fig. 3c). σ_max_ showed a sex effect only (*p* = 0.034), with females higher overall (Fig. 3d). Young’s modulus likewise showed a sex effect only (*p* < 0.001), with females higher overall; no dose or interaction effects were detected (Fig. 3e).

### Growth plate and metaphyseal histomorphometry

**Fig. 4:**
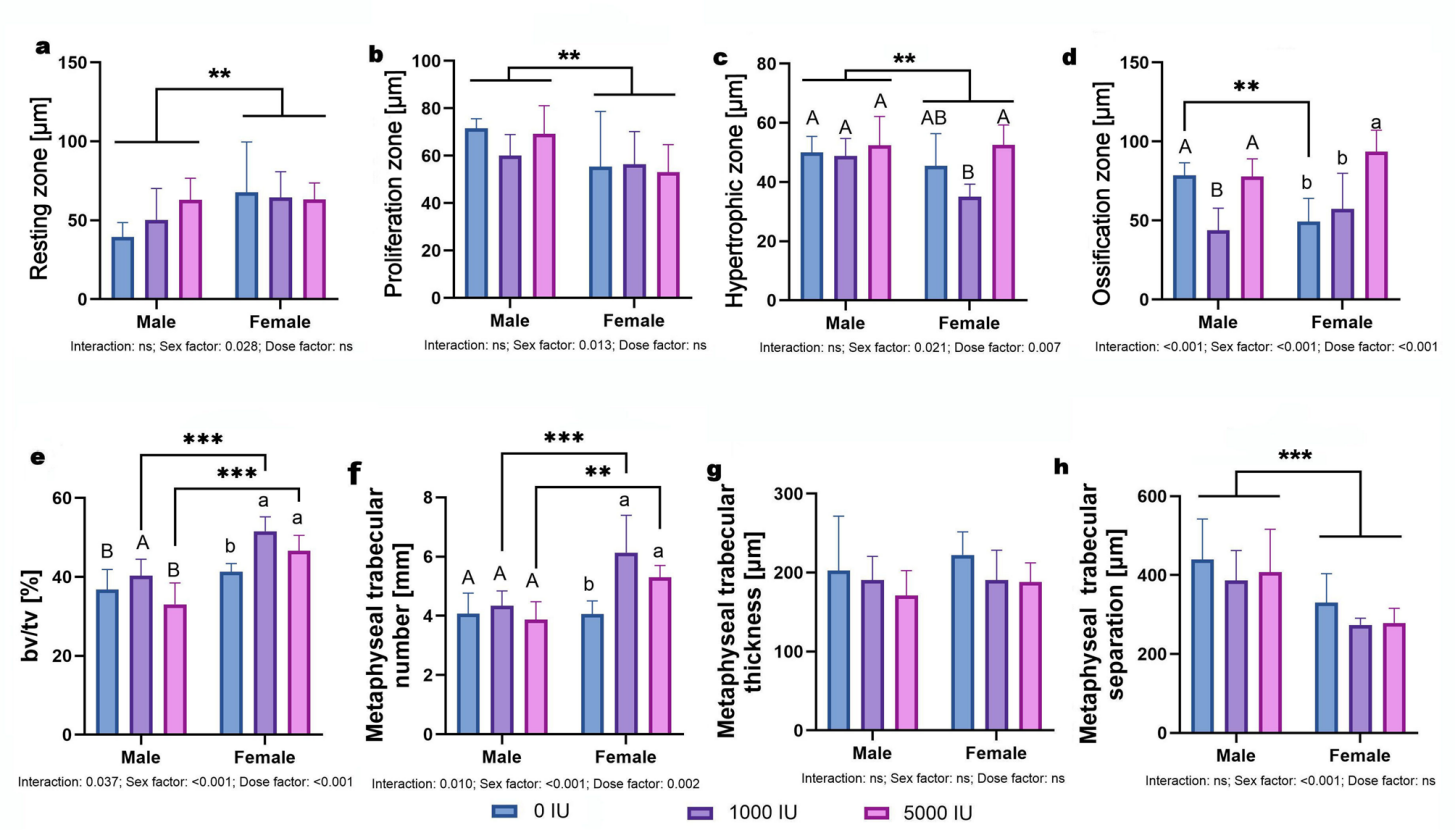
Effects of dietary vitamin D supplementation on bone plate and metaphyseal morphometry in male and female Wistar rats. Animals were assigned to one of three dietary groups: 0 IU/kg (Group I), 1000 IU/kg (Group II), or 5000 IU/kg (Group III) of vitamin D₃ (*n* = 6 per sex per group): (**a**) resting zone [Zone I], (**b**) proliferation zone [Zone II], (**c**) hypertrophic zone [Zone III], (**d**) ossification zone [Zone IV] (**e**) bone volume to total volume ratio, (**f**) metaphyseal trabecular number, (**g**) metaphyseal trabecular thickness and (**h**) metaphyseal trabecular separation. Data are presented as mean ± SEM. Statistical analyses were conducted in GraphPad Prism (version 10.5.0) using two-way ANOVA to assess the effects of vitamin D dose, sex, and their interaction. Statistical significance was set at *p* < 0.05. Capital letters indicate significant differences between dietary doses within the sex groups; brackets denote significant differences between sexes; and asterisks (*) represent p-values (**p < 0.01; ***p < 0.001).

Zone I showed sex effect only (*p* = 0.028); no dose or interaction. Females had wider Zone I overall; pairwise significance was evident in the deficient group (Fig. 4a). Zone II showed sex effect only (*p* = 0.013); no dose or interaction. Males had wider Zone II overall (Fig. 4b). Zone III: main effects of sex (*p* = 0.021) and dose (*p* = 0.007) without interaction. Females exceeded males overall, and pooled across sex the 5000 IU/kg group was greater than 0 and 1000 IU/kg (Fig. 4c). Zone IV showed significant interaction (*p* < 0.001), with simple effects showing that within males the 1000 IU/kg group had a thinner Zone IV than 0 and 5000 IU/kg (both *p* < 0.001), within females the 5000 IU/kg group exceeded 0 and 1000 IU/kg (*p* < 0.001), and between sexes at 0 IU/kg males > females (*p* < 0.01) (Fig. 4d). BV/TV showed interaction (*p* = 0.037) with sex and dose effects (both *p* < 0.001). Females > males at 1000 and 5000 IU/kg (*p* < 0.001); within males, 1000 IU/kg > 0 and 5000 IU/kg (*p* = 0.016 and *p* < 0.01); within females, 0 IU/kg < 1000 and 5000 IU/kg (*p* < 0.001) (Fig. 4e). Trabecular number showed interaction (*p* = 0.010), sex (*p* < 0.001) and dose (*p* = 0.002) effects. Females > males at 1000 IU/kg (*p* < 0.001) and 5000 IU/kg (*p* < 0.01); within females, 0 IU/kg < 1000 and 5000 IU/kg (*p* < 0.001); no dose differences within males (Fig. 4f). Trabecular thickness showed no effects of sex, dose, or interaction (Fig. 4g). Trabecular separation showed sex effect only (*p* < 0.001); no dose or interaction. Males showed greater separation across groups (Fig. 4h).

### Macro- and microelements

**Fig. 5:**
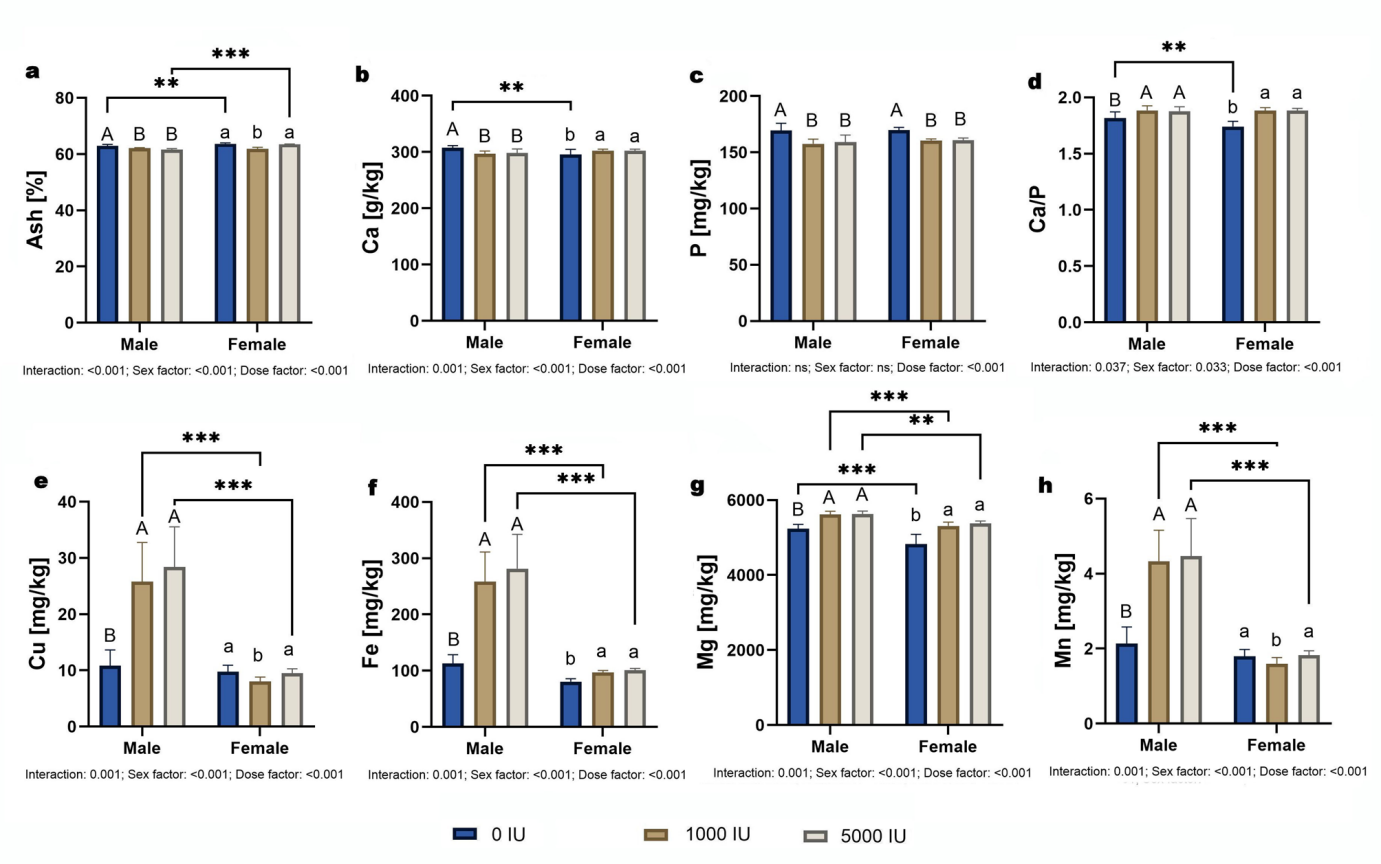
Effects of dietary vitamin D supplementation on macro- and microelement content in male and female Wistar rats. Animals were assigned to one of three dietary groups: 0 IU/kg (Group I), 1000 IU/kg (Group II), or 5000 IU/kg (Group III) of vitamin D₃ (*n* = 6 per sex per group): (**a**) Ash percentage, (**b**) Calcium, (**c**) Phosphorus, (**d**) Calcium-to-phosphorus ratio, (**e**) Copper, (**f**) Iron, (**g**) Magnesium and (**h**) Manganese. Data are presented as mean ± SEM. Statistical analyses were conducted in GraphPad Prism (version 10.5.0) using two-way ANOVA to assess the effects of vitamin D dose, sex, and their interaction. Statistical significance was set at *p* < 0.05. Capital letters indicate significant differences between dietary doses within the sex groups; brackets denote significant differences between sexes; and asterisks (*) represent p-values (**p < 0.05; ***p < 0.001).

Ash content showed a dose×sex interaction (*p* < 0.001). Simple effects: within males, 0 IU/kg > 1000 and 5000 IU/kg (*p* = 0.014 and *p* < 0.001); within females, 1000 IU/kg < 0 and 5000 IU/kg (both *p* < 0.001). Between sexes, males > females at 0 and 5000 IU/kg (*p* < 0.001) (Fig. 5a). Calcium also showed an interaction (*p* = 0.001). Within males, 0 IU/kg > 1000 and 5000 IU/kg (*p* = 0.007 and *p* = 0.024); within females, no dose differences. Between sexes, males > females at 0 IU/kg only (*p* < 0.01) (Fig. 5b). Phosphorus showed a dose effect only (*p* < 0.001), with 0 IU/kg > 1000 and 5000 IU/kg in both sexes; no sex or interaction effects (Fig. 5c). The Ca/P ratio showed an interaction (*p* = 0.037). Within each sex, 0 IU/kg < 1000 and 5000 IU/kg (*p* < 0.001); between sexes, males > females at 0 IU/kg (*p* < 0.01) (Fig. 5d). Copper, iron, and manganese each showed dose×sex interactions (*p* = 0.001). Within males, 0 IU/kg < 1000 and 5000 IU/kg for all three elements (all *p* < 0.001); within females, no dose differences. Between sexes, males > females at 1000 and 5000 IU/kg (all *p* < 0.001) (Fig. 5e, f,h). Magnesium also showed an interaction (*p* = 0.001): within both sexes, 0 IU/kg < 1000 and 5000 IU/kg (*p* < 0.001); between sexes, males > females across doses (*p* < 0.001) (Fig. 5g).

### X-ray diffraction analysis

**Fig. 6:**
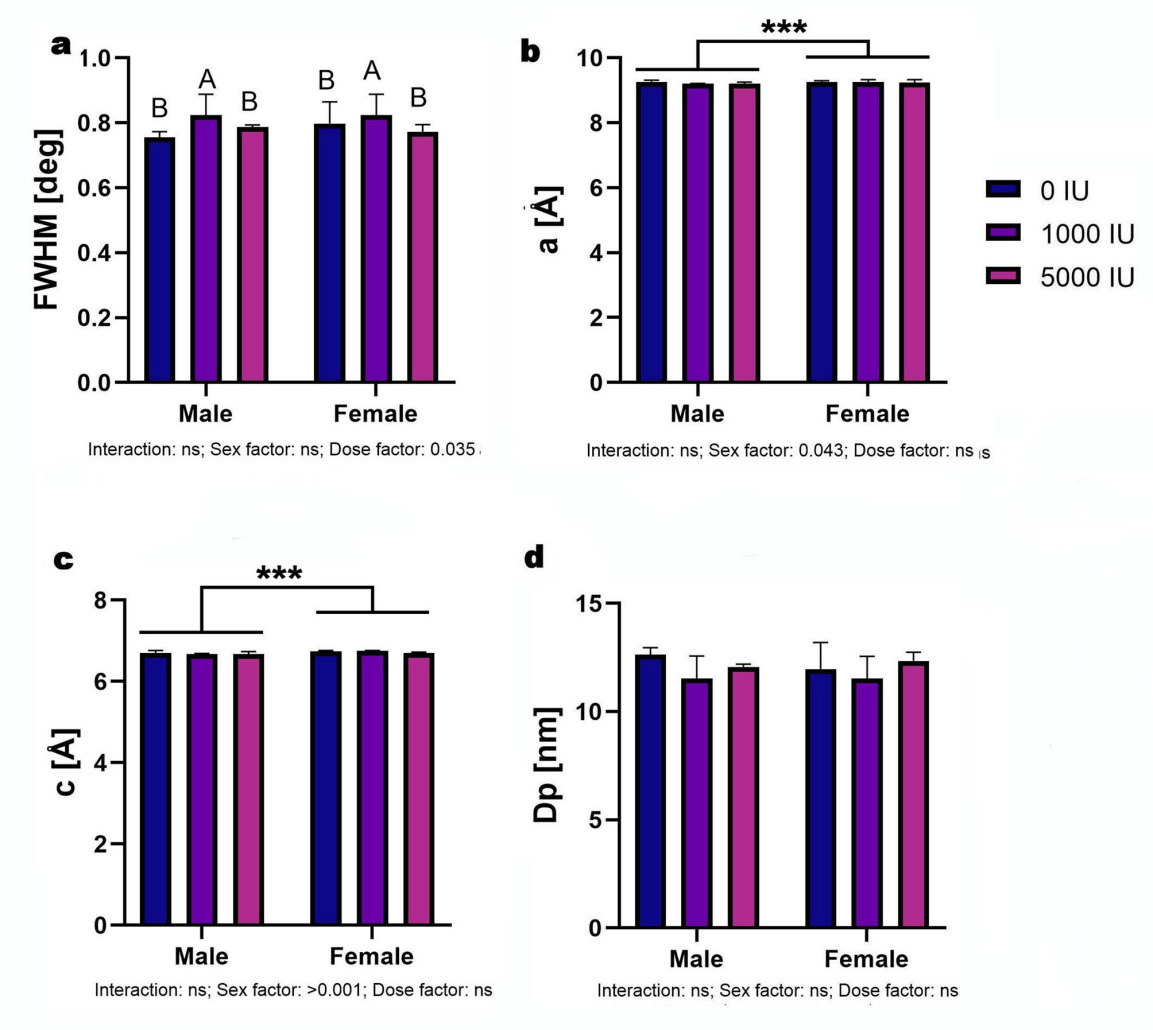
Effects of dietary vitamin D supplementation on X-ray Diffraction in male and female Wistar rats. Animals were assigned to one of three dietary groups: 0 IU/kg (Group I), 1000 IU/kg (Group II), or 5000 IU/kg (Group III) of vitamin D₃ (*n* = 6 per sex per group): (**a**) full width at half maximum, (**b**) “a” lattice parameter, (**c**) “c” lattice parameter and (**d**) crystallite size. Data are presented as mean ± SEM. Statistical analyses were conducted in GraphPad Prism (version 10.5.0) using two-way ANOVA to assess the effects of vitamin D dose, sex, and their interaction. Statistical significance was set at *p* < 0.05. Capital letters indicate significant differences between dietary doses within the sex groups; brackets denote significant differences between sexes; and asterisks (*) represent p-values (***p < 0.001).

In the analysis of FWHM, no interaction was observed; however, a significant dose effect was present (*p* = 0.035). Within males, the 1000 IU group displayed significantly higher FWHM values compared to both the deficiency and 5000 IU groups (Fig. 6a; *p* = 0.046). No significant differences were observed among female groups. For the *a* lattice parameter, only the sex factor was statistically significant (*p* = 0.043). Specifically, in the 1000 IU group, females exhibited significantly higher *a* values than males (Fig. 6b; *p* < 0.001). Analysis of the *c* lattice parameter also revealed a significant sex effect (*p* < 0.001). In the 1000 IU group, females had significantly higher *c* values than males (Fig. 6c; *p* < 0.001). No statistically significant differences were detected in the Dp across any groups, regardless of sex or dietary treatment (Fig. 6d).

### Bone markers immunoexpression

**Fig. 7:**
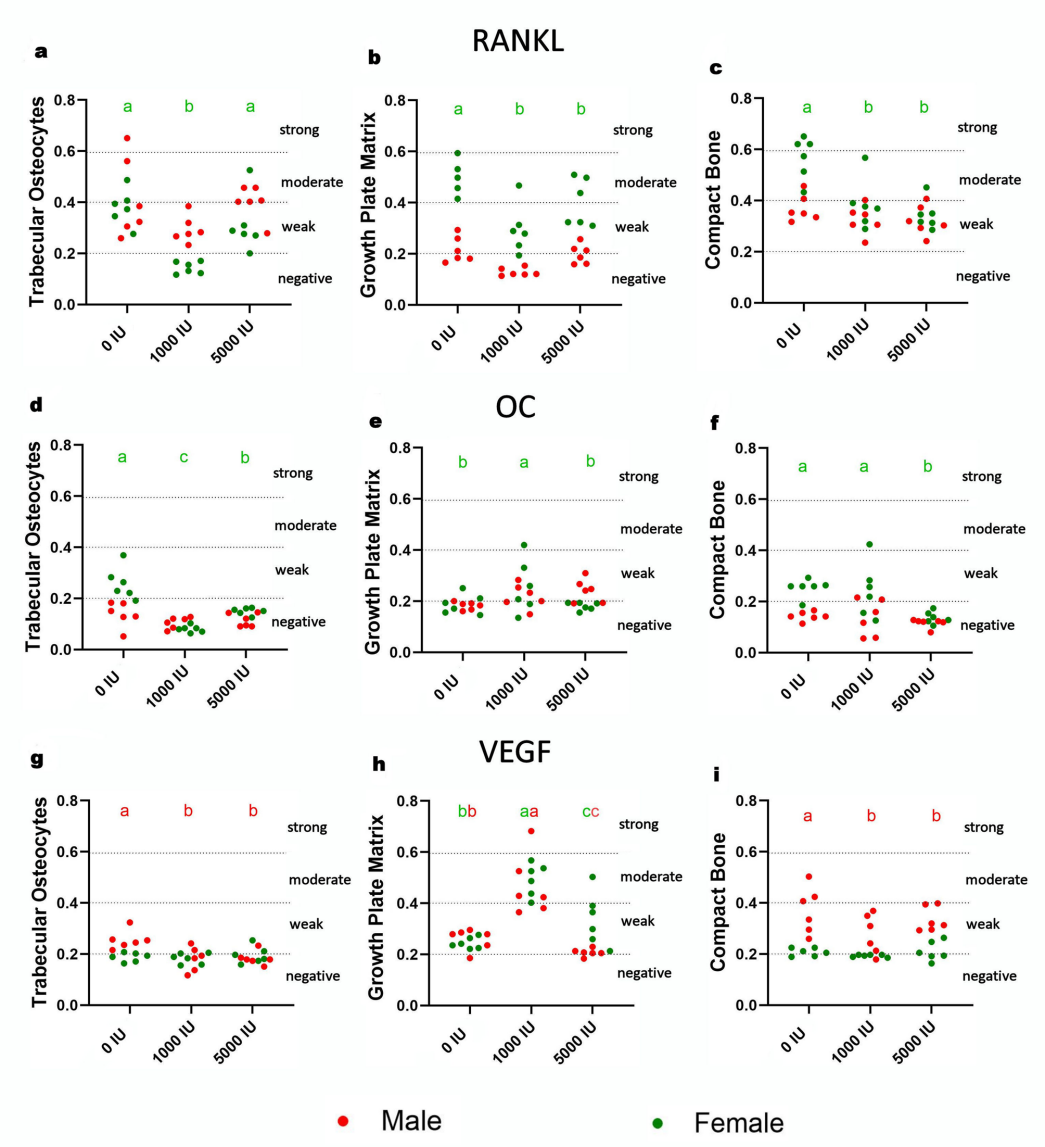
Effects of dietary vitamin D supplementation on immunoexpression of bone metabolism markers in male and female Wistar rats. Rats were assigned to one of three dietary groups: 0 IU/kg (Group I), 1000 IU/kg (Group II), or 5000 IU/kg (Group III) vitamin D (*n* = 6 per sex per group). (**a**) RANKL in trabecular bone osteocytes, (**b**) RANKL in growth plate matrix, (**c**) RANKL in compact bone, (**d**) Osteocalcin in trabecular bone osteocytes, (**e**) Osteocalcin in growth plate matrix, (**f**) Osteocalcin in compact bone, (**g**) VEGF in trabecular bone osteocytes, (**h**) VEGF in growth plate matrix, and (**i**) VEGF in compact bone. Statistical analyses were conducted in GraphPad Prism (version 10.5.0) using two-way ANOVA to assess the effects of vitamin D dose, sex, and their interaction. Statistical significance was set at *p* < 0.05. Red letters show statistical differences between doses in males, green in females.

Immunoexpression outcomes were compartment- and sex-specific (Fig. 7a–i). For RANKL, changes were confined to females: in trabecular osteocytes the 1000 IU/kg group was lower than 0 and 5000 IU/kg (*p* = 0.009), and in the growth-plate matrix and compact bone both supplemented groups were reduced versus deficiency; males showed no differences. For osteocalcin, significant effects again appeared only in females: trabecular osteocytes were reduced in both supplemented groups (greater reduction at 1000 IU/kg), the growth-plate matrix increased at 1000 IU/kg (*p* < 0.001), and compact bone decreased at 5000 IU/kg (*p* = 0.007); males were unchanged. For VEGF, males showed reductions in trabecular osteocytes at both supplemented doses (*p* = 0.001 and 0.002), the growth-plate matrix exhibited a biphasic pattern in both sexes (increase at 1000 IU/kg, decrease at 5000 IU/kg vs. 0 IU/kg; *p* < 0.001 in males and *p* = 0.002 in females), and compact bone decreased in males at both supplemented doses (*p* = 0.018 and *p* < 0.001) with no differences in females.

### Serum biomarkers analysis

Serum biomarkers showed selective, sex-specific dose effects (Table 1). GH showed no dose effect, with a consistent sex difference (males > females). Osteocalcin increased at both supplemented doses in males (*p* = 0.011) and was biphasic in females (increase at 1000 IU/kg, *p* = 0.005; decrease at 5000 IU/kg, *p* < 0.001). OPG decreased in males at both supplemented doses (*p* < 0.001) and in females at 1000 IU/kg only (*p* = 0.019). RANKL increased in males at 1000 IU/kg (*p* = 0.048) and decreased in females at both supplemented doses (*p* = 0.033 and *p* = 0.039). IGF-1, IL-1α, IL-6, and BALP showed no dose effects in either sex. TNF-α rose in males at 1000 IU/kg (*p* = 0.005) but was biphasic in females (decrease at 1000 IU/kg, *p* = 0.006; increase at 5000 IU/kg, *p* < 0.001). The RANKL/OPG ratio was highest in males at 1000 IU/kg (vs. 0 and 5000 IU/kg, *p* < 0.001); in females, 5000 IU/kg yielded the lowest ratio (vs. 0 and 1000 IU/kg, *p* = 0.012 and *p* = 0.017), with the male 1000 IU/kg group exceeding all female groups (*p* = 0.008).

**Table 1:** Serum concentrations of bone metabolism and inflammatory biomarkers in male and female Wistar rats following dietary vitamin D supplementation.

	Male			Female		
	0 IU	1000 IU	5000 IU	0 IU	1000 IU	5000 IU
GH, ng/ml	10.73 ± 1.08^a^	8.90 ± 1.61^a^	10.62 ± 2,19^a^	4.48 ± 1.06^b^	5.63 ± 1.09^b^	5.96 ± 1.25^b^
OC, ng/ml	13.53 ± 4.40^c^	20.08 ± 6.59^b^	17.57 ± 2.70^b^	26.93 ± 8.87^b^	30.44 ± 6.81^a^	13.22 ± 3.82^c^
OPG, pg/ml	549.96 ± 84.98^a^	479.13 ± 74.80^b^	509.08 ± 136.39^b^	443.49 ± 133.82^b^	347.29 ± 66.07^c^	452.36 ± 90.54^b^
RANKL, pg/ml	36.78 ± 8.99^b^	63.34 ± 16.86^a^	49.67 ± 15.62^b^	53.65 ± 20.41^a^	40.71 ± 15.22^b^	35.42 ± 10.07^b^
IGF-1, ng/ml	921.77 ± 115.57	884.64 ± 191.21	1067.35 ± 150.89	953.70 ± 100.35	982.55 ± 73.12	887.32 ± 197.15
IL-1, pg/ml	20.05 ± 4.46	17.53 ± 1.09	19.23 ± 3.64	18.45 ± 1.74	19.57 ± 1.13	19.39 ± 0.43
IL-6, pg/ml	23.16 ± 4.49	18.90 ± 5.35	23.29 ± 5.83	18.72 ± 3.46	18.89 ± 6.33	18.95 ± 1.91
IL-10, pg/ml	41.15 ± 6.46^b^	84.14 ± 12.34^a^	39.50 ± 5.02^b^	16.83 ± 9.16^c^	15.90 ± 5.07^c^	35.25 ± 5.71^b^
TNFα, pg/ml	43.48 ± 8.41^a^	32.85 ± 3.03^b^	39.89 ± 5.87^a^	28.21 ± 2.50^c^	22.26 ± 1.04^d^	32.71 ± 2.49^b^
BALP, ng/ml	8.20 ± 2.52	9.41 ± 3.26	9.27 ± 3.09	6.89 ± 0.26	8.94 ± 3.29	9.38 ± 3.32
RANKL/OPG	0.067 ± 0.01^c^	0.132 ± 0.03^a^	0.098 ± 0.01^c^	0.118 ± 0.01^b^	0.115 ± 0.03^b^	0.079 ± 0.01^c^

Data are shown as mean ± SEM with statistical significance set at *p* < 0.05. Different lowercase letters indicate statistically significant differences between groups. GH - Growth Hormone, OC - Osteocalcin, OPG – Osteoprotegrin, RANKL - Receptor Activator of Nuclear Factor κB Ligand, IGF-1 - Insulin-like Growth Factor 1, IL-1 – Interleukin 1, IL-6 – Interleukin 6, IL-10 – Interleukin 10, TNFα - Tumor Necrosis Factor alpha, BALP - Bone-specific Alkaline Phosphatase.

## Discussion

This study examined how dietary supplementation with vitamin D at two concentrations (1000 and 5000 IU/kg) influences bone development and quality in male and female Wistar rats. The analysis encompassed a comprehensive evaluation of skeletal parameters, including bone geometry, mechanical performance, microarchitecture, mineral composition, serum biomarkers, immunohistochemical reactivity of bone-regulating proteins, and crystallographic properties. The results revealed distinct dose- and sex-dependent effects of vitamin D on skeletal structure and function, highlighting beneficial outcomes at moderate supplementation and a more complex, often attenuated response at higher doses.

The increase in bone mass observed in males supplemented with 1000 IU/kg of vitamin D, without corresponding changes in body weight or bone length, suggests a targeted skeletal effect rather than general somatic growth. Enhanced bone mass in the context of stable somatic parameters implies improved skeletal accrual efficiency. In both sexes, this translated into significantly higher BMD in the supplemented groups compared to the deficiency group, though without parallel increases in bone length or the Seedor index. Since BMD is frequently used as a surrogate for bone strength^28^, this elevation may indicate enhanced mineral incorporation or improved matrix quality. However, the unchanged Seedor index, a geometric density proxy suggests that the observed BMD improvements did not reflect changes in bone size or volume. This disconnect supports prior observations that BMD alone does not fully capture mechanical competence^29–31^, highlighting the importance of evaluating both material and structural properties when assessing bone health.

Vitamin D’s influence extended to biomechanical behavior, particularly in the parameters of F_max_ and W_max_. In males, both the 1000 IU/kg and 5000 IU/kg groups demonstrated significantly higher F_max_ compared to the deficiency group, indicating improved load-bearing capacity. However, for W_max_, a significant increase was observed only in the 1000 IU group, suggesting that moderate supplementation enhanced the bone’s ability to absorb energy before failure. These mechanical improvements coincided with increases in bone mass and BMD, reinforcing the role of moderate vitamin D intake in improving functional bone traits. Stiffness, on the other hand, remained unaffected by dietary treatment, indicating that vitamin D may selectively enhance strength and plasticity without altering rigidity. However, it is important to note that in skeletal tissue, plastic deformation does not result in elongation but rather initiates microcrack formation and repeated loading within the plastic region may lead to cumulative microdamage and eventual fatigue failure, manifesting as stress fractures. This is particularly relevant given no increase in Young’s modulus observed in supplemented males, which may indicate a shift toward more compliant but damage-prone tissue. This pattern aligns with previous findings^12,32^ where mechanical gains occurred independently of changes in stiffness or bone geometry. Lu et al. (2019) similarly showed that local material properties, rather than global shape, better predict mechanical performance^33^. Interestingly, these advantages were more pronounced in males. Despite similar increases in BMD, females did not show significant changes in F_max_ or W_max_, suggesting potential influences of sex-specific regulatory pathways. The absence of dietary effects on CSA and Ix further supports the interpretation that vitamin D’s impact is primarily exerted through changes in tissue composition and mineralization rather than geometry^34^. Moreover, the lack of dietary effect on CSA and Ix further supports the conclusion that vitamin D acts primarily on material properties and mineralization rather than altering bone geometry under these experimental conditions, as both parameters reflect bone size and structural distribution, which remained unchanged despite improvements in mechanical performance^35^.

Analysis of bone material properties provided deeper insights into the quality of the bone matrix. A significant increase in εmax was observed in both sexes at 1000 IU/kg, indicating enhanced toughness and deformability, critical traits for resisting brittle fractures. These gains in plasticity, without corresponding changes in stiffness or bone geometry, point toward modifications in the collagen–mineral matrix that governs mechanical behavior at the tissue level. While Young’s modulus was not significantly influenced by vitamin D dose, sex differences were evident, with females generally showing higher values than males in both the deficiency and high-dose groups. Ash content also showed a sex- and dose-dependent response: in males, ash percentage was highest in the deficiency group, while in females, the 1000 IU group had the lowest mineral content. This pattern suggests that vitamin D may shift the organic-to-mineral ratio, potentially favoring collagen-rich matrix properties over pure mineral-driven stiffness, particularly at moderate doses. These findings are consistent with vitamin D’s known role in promoting collagen maturation and cross-linking, as shown in previous work^36–38^. A comparable pattern was described previously, demonstrating that vitamin D sufficiency mitigates bone loss and excessive osteoclastogenesis, reinforcing the concept that moderate vitamin D levels can preserve bone function through qualitative improvements, even in the absence of gains in stiffness^6,39^. Although both sexes showed mechanical benefits at 1000 IU, females displayed a consistently higher Young’s modulus and did not exhibit the same pattern of mineral reduction, indicating possible sex-specific differences in matrix adaptation^5,40,41^. These sex-specific responses highlight the importance of considering hormonal context when evaluating the effects of nutritional interventions on skeletal integrity. Taken together, because ash and calcium values are normalized to dry mass, they primarily reflect the mineral-to-organic ratio rather than whole-bone mineral quantity; a higher ratio can increase brittleness, so these apparent material changes warrant cautious interpretation and motivate direct, sex-stratified assays of matrix composition and bone-mineral quality in future work.

In vitamin D-deficient animals, we observed hallmark features of impaired endochondral ossification, most notably a widened and disorganized growth plate, particularly within the hypertrophic zone. These morphological abnormalities are indicative of delayed chondrocyte maturation and mineralization failure, resulting in the accumulation of hypertrophic cells that fail to transition into bone, a classic presentation of early rickets-like pathology, a pattern consistent with established descriptions of rickets pathology^4,42^. The findings align with those of Verlinden and Carmeliet (2021), who noted disrupted chondrocyte apoptosis and ossification under vitamin D-deficient conditions, and further supported by Idelevich et al. (2011), who demonstrated that 1,25(OH)₂D₃ deficiency impairs growth plate progression even in rats with normal renal function^43,44^. Conversely, rats supplemented with 1000 IU/kg of vitamin D, particularly females, showed a markedly thinner hypertrophic zone, suggesting effective chondrocyte turnover and ossification. This was paralleled by improvements in mechanical and material bone properties discussed earlier, indicating a coordinated enhancement of both endochondral and matrix-level bone formation at this dosage. Interestingly, the 5000 IU/kg group also exhibited a widened hypertrophic zone in females, despite preserved bone mass and geometry. This suggests that excessive vitamin D exposure may subtly disrupt the terminal maturation of hypertrophic chondrocytes, potentially through altered matrix remodeling or vascular signaling. Supporting this, Lin et al. (2002) demonstrated that supraphysiologic doses of 1,25(OH)₂D₃ could alter VEGF immunoexpression and interfere with chondrocyte dynamics at the chondro-osseous junction^45^.

Sex-specific differences in trabecular architecture were prominent, particularly in trabecular number, which was significantly higher in female rats receiving either 1000 IU or 5000 IU of vitamin D compared to their male counterparts. This pattern suggests a more robust trabecular remodeling response in females, even at higher supplementation levels. Despite these changes, trabecular thickness remained unchanged across all groups, indicating that modifications in trabecular bone volume were primarily due to increased trabecular number rather than expansion in trabecular width. The highest BV/TV was observed in the 1000 IU female group, while the deficiency group had the lowest values within each sex. This nonlinear trend suggests that while moderate vitamin D supplementation promotes trabecular accrual, further increases in serum 25(OH)D beyond physiological levels do not confer additional benefit in healthy growing animals. These results differ from the linear dose-response reported by Anderson et al. (2008), highlighting the context-dependence of vitamin D action^6^. In males, 1000 IU supplementation led to improved BV/TV compared to both deficient and high-dose groups, but the overall magnitude of change was less pronounced than in females. Additionally, trabecular separation was consistently greater in males than females across all dietary groups, further underscoring sexual dimorphism in skeletal microarchitecture and adaptive potential.

XRD analysis revealed subtle yet informative changes in apatite lattice structure. A significant increase in FWHM was observed in the 1000 IU male group, suggesting reduced crystallite size or increased lattice disorder in this cohort. Additionally, the a-axis parameter was significantly higher in 1000 IU females, while the c-axis parameter was elevated in the same group relative to males, indicating potential sex- and dose-dependent variations in unit cell geometry. Although Dp values remained unchanged, these isolated lattice shifts likely reflect local substitutions within the hydroxyapatite crystal lattice. Specifically, increased incorporation of divalent cations such as magnesium and manganese, both elevated in supplemented male groups, may partially substitute for calcium, inducing lattice compression and decreasing crystallite size, a phenomenon well-documented in bone mineral chemistry^46–48^. Complementary shifts in Ca/P ratio, particularly the significant increase in supplemented females, support this interpretation. A Ca/P ratio exceeding the stoichiometric value of 1.67 for pure hydroxyapatite suggests the presence of carbonate-substituted apatite (B-type) or additional calcium phases like surface CaCO₃ deposits, as previously reported under high dietary calcium conditions. This aligns with the elevated calcium levels observed in males, potentially reflecting higher feed intake and body mass. Such substitutions not only affect crystal structure but may also enhance mechanical behavior, a hypothesis corroborated by the observed increases in F_max_ in the 1000 IU male group, where mineral maturation and compactness coincided with improved load-bearing capacity. Collectively, these findings reinforce the view that vitamin D modulates bone mineral properties not just by quantity, but through qualitative alterations in crystal architecture, with implications for both stiffness and toughness. These mechanisms mirror earlier descriptions by Wopenka and Pasteris (2005), who emphasized the critical link between apatite lattice substitutions and biomechanical integrity of bone tissue^49^.

Bone marker immunoexpression and angiogenic signaling revealed additional compartment- and sex-specific effects. Females exhibited reduced RANKL immunoexpression in trabecular osteocytes and a lower RANKL/OPG ratio, especially at higher doses, suggesting suppression of osteoclastogenesis and bone resorption^50^. OC immunoexpression declined in most bone compartments, but was upregulated in the 1000 IU growth plate matrix, potentially reflecting localized stimulation of endochondral ossification. In males, the 1000 IU dose increased RANKL and OC serum levels while decreasing OPG, producing a higher RANKL/OPG ratio, indicating enhanced resorptive activity at this dose. These divergent responses are further supported by TNF-α dynamics, which increased in 1000 IU males but decreased in females, reinforcing the sex- and dose-specific nature of immune-bone interactions. Notably, Anderson et al. (2008) reported an inverse relationship between serum 25(OH)D levels and RANKL/OPG mRNA immunoexpression in whole bone^6^. Our findings partially align with this observation: in females, vitamin D supplementation lowered RANKL and the RANKL/OPG ratio, suggesting a protective, anti-resorptive effect. In contrast, the male response to moderate supplementation appeared to enhance resorptive signaling, potentially reflecting a lower threshold for activation or fundamentally distinct regulatory pathways governing bone remodeling.

A brief note on dose context is warranted for interpretation and translation. In this model, 0 IU/kg diet operationalizes vitamin D deficiency, 1,000 IU/kg corresponds to the established AIN-93G adequacy for growing rats, and 5,000 IU/kg represents a supraphysiological but non-toxic intake used to probe effects beyond adequacy under calcium-replete conditions. Human recommendations are expressed as fixed daily intakes rather than per-kilogram dosing, typically 600–800 IU per day in adults with an upper intake level of 4,000 IU per day; using rough intake scaling, 1,000 IU/kg feed in rats aligns with standard human supplementation, whereas 5,000 IU/kg approximates a higher supplemental range on the order of 2,800–3,000 IU per day and remains below the adult upper limit. For veterinary translation to swine, practical formulations generally include about 200–800 IU vitamin D per kilogram of feed depending on class and physiological state; thus 1,000 IU/kg is near physiologic practice, while 5,000 IU/kg is well above husbandry targets and should be viewed as an experimental probe rather than a feeding recommendation. These comparisons are heuristic rather than direct dose conversions, but they situate our findings: adequacy at 1,000 IU/kg reflects physiologic supplementation, 0 IU/kg models deficiency, and 5,000 IU/kg tests the upper range of response without implying routine use in humans or livestock^51,52^.

Taken together, our findings confirm that dietary vitamin D deficiency during late growth exerts clear detrimental effects on bone development, particularly by impairing mineralization, endochondral ossification, and tissue-level mechanical behavior. Moderate supplementation at 1000 IU/kg yielded the most consistent skeletal benefits, enhancing both material and functional bone traits, especially in males. However, increasing the dose to 5000 IU/kg did not consistently confer further advantages. In several mechanical and histological parameters, outcomes at this higher dose were comparable to those seen in deficient animals, suggesting a plateau beyond which additional vitamin D may not translate into further improvement. While 5000 IU/kg cannot be considered a pharmacological megadose, it may represent the upper bound of physiological supplementation under normal dietary calcium and phosphorus conditions. Notably, prior studies using much higher doses, such as 10,000 IU/kg in mice (Williamson et al., 2017) or 40,000 IU/kg in older rats (Bhamb et al., 2017), have reported variable outcomes ranging from mild skeletal benefit to neutral effects, reinforcing the context-specific nature of vitamin D responsiveness^10,12^. Bhargava et al. (2024) demonstrated that elevated vitamin D signaling may modulate vascular and skeletal mineralization mechanisms, highlighting a broader systemic sensitivity to high-dose exposure^53^. Our data support the concept of a biphasic or plateau response curve for vitamin D, emphasizing that more is not always better and that optimized, context-aware dosing is essential for maintaining bone health during growth.

### Novelty

Our dietary design leveraged the AIN-93G standard, in which 1,000 IU/kg vitamin D₃ is considered adequate for growth. Consequently, the observation that 0 IU/kg (vitamin D deficiency) impaired skeletal outcomes and that 1,000 IU/kg performed best relative to deficiency was expected a priori. The purpose here was not to re-establish adequacy but to quantify the dose-response across multiple hierarchical levels of bone quality and to determine sex-specific sensitivities during late growth, while also testing whether a supraphysiological dose (5,000 IU/kg) confers benefits beyond the standard level. In this context, our novelty lies in integrating densitometry, whole-bone mechanics (including post-yield behavior), mineral/elemental profiling, hydroxyapatite lattice features by X-ray diffraction, and growth-plate zone–resolved histomorphometry with immunoexpression, revealing that improvements at 1,000 IU/kg extend beyond mineral accrual to material and mechanical properties, sex is a major determinant of effect magnitude, and 5,000 IU/kg increases systemic 25(OH)D without consistent skeletal gains over 1,000 IU/kg.

## Data Availability

All data generated or analyzed during this study are included in this published article.
